# Farther Remarks on the Useless State of the Lower Limbs, in Consequence of a Curvature of the Spine, Being a Supplement to a Former Treatise on That Subject

**Published:** 1782

**Authors:** 


### THE LONDON MEDICAL JOURNAL,

### For JULY, AUGUST, and SEPTEMBER, 1782.

### SECTION I.

### BOOKS.

I. *Farther remarks on the useless state of the lower limbs, in consequence of a curvature of the spine, being a supplement to a former treatise on that subject. By* Percival Pott, F. R. S. *Surgeon to St. Bartholomew's Hospital.* 8vo. Johnson, *London,* 1782. 64 pages.

IT is now three years since this ingenious and respectable writer first published his observations on the disease which makes the subject of the present tract. His wishes and expectations with regard to the method of cure, which he then proposed, have been most pleasingly fulfilled. He has received such repeated testimony of its success from so large a number of the moft eminent practitioners, not only in this town and kingdom, but in many other parts of Europe; that thefe, he affures us, added to his own experience, have completely fatisfied him, and enabled him to fay, that in proper cafes, and under proper treatment, he has no doubt of its being univerfal.

The difeafe in queftion, is a difeafe of the Spine, producing an alteration in its natural figure, and not unfrequently attended with a partial, or a total lofs of the power of ufing, or even of moving, the lower limbs.

From this laft circumftance (the lofs of the ufe of the limbs) it has in general been called a palfy, and treated as a paralytic affection; to which it is in almoft every refpect perfectly unlike.

The occafion of this miftake, our author obferves, is palpable; the patient is deprived of the ufe of his legs, and has a deformed incurvation of the fpine; the incurvation is fuppofed to be caufed by a diflocation of the vertebræ; the difplaced bones are thought to make an unnatural preffure on the fpinal marrow, and a preffure on that being very likely to produce a paralyfis of fome kind, the lofs of the ufe of the legs is in this cafe determined to be fuch:

the the truth is, that there is no diflocation, no unnatural preffure made on the fpinal marrow, nor are the limbs by any means paralytic, as will appear to whoever will examine the two complaints with any degree of attention.

In the true paralyfis the mufcles of the affected limb are foft, flabby, unrefifting, and incapable of being put into even a tonic ftate; the limb itfelf may be placed in almoft any pofition, and the joints are perfectly and eafily moveable in every direction.

In the prefent cafe, the mufcles are more extenuated, and leffened in fize, but they are rigid, and always at leaft in a tonic ftate; by which the knees and ancles acquire a ftiffnefs not very eafy to overcome; by means of this ftiffnefs, mixed with a kind of fpafm, the legs of the patient are either conftantly kept ftretched out ftrait, in which cafe confiderable force is required to bend the knees, or they are by the action of the ftronger mufcles drawn acrofs each other, in fuch manner as to require as much to feparate them; when the leg is in a ftrait pofition, the extenfor mufcles act fo powerfully as to require a confiderable degree of force to bend the joints of the knees; and when they have been bent, the legs are immediately drawn up with the heels towards the buttocks: by the rigidity of the ancle joints, joined to the ſpaſmodic action of the gaſtrocnemii muſcles, the patient's toes are pointed downward in ſuch manner as to render it impoſſible for him to put his foot flat to the ground; which makes one of the deciſive characteriſtics of the diſtemper.

Theſe, according to our author, are the marks of the diſtinction which ought to be made between the two diſeaſes. They are certainly fully ſufficient to ſhew the impropriety of confounding them with each other.

The majority of thoſe who labour under this diſeaſe are infants or young children. Adults are by no means exempt from it; but Mr. Pott has never ſeen it at an age beyond forty.

When it attacks a child who is old enough to have walked properly, its aukward and imperfect manner of uſing its legs is the circumſtance which firſt excites attention, and the incapacity of uſing them at all, which very ſoon follows, fixes that attention and alarms the friends.

The account moſt frequently given is, that for ſome time previous to the incapacity, the child had been obſerved to be languid, liſtleſs, unwilling to move much, or briſkly, and that he was very ſoon tired; that he had been obſerved ferved frequently to trip and ftumble, although no impediment lay in his way; that when he moved haftily, his legs would crofs each other involuntarily, by which he was often and fuddenly thrown down; that if he endeavoured to ftand ftill, and upright, unfupported by another perfon, his knees would totter and bend under him; that he could not, with any degree of precifion or certainty, fteadily direct either of his feet to any particular point; that foon after this he complained of frequent pains and twitchings in his thighs, particularly when in bed, and of an uneafy fenfation at the pit of his ftomach; that when he fat on a chair, his legs were almoft always found acrofs each other, and drawn up under the feat; and that in a little time after thefe particulars had been obferved, he totally loft the power of walking.

Thefe, we are told, are the general circumftances which are found, at leaft in fome degree, and that pretty uniformly, in moft infants and children; but there are others which are different in different fubjects.

If the incurvation be of the neck, and feveral vertebræ are affected, the child finds it painful to fupport its own head. If the dorfal vertebræ are difeafed, lofs of appetite, hard dry cough, laborious laborious refpiration, quick pulfe, and difpofition to hectic, appear pretty early. In an adult, the attack and the progrefs of the difeafe are much the fame; but there are fome few circumftances, our author obferves, which may be learned from a patient of fuch age, which either do not make an impreffion on a child, or do not happen to it.

An adult, in a cafe where no violence hath been committed, or received, will tell you, that his firft intimation was a fenfe of weaknefs in his back bone, accompanied with a heavy, dull kind of pain and great laffitude; that this was foon followed by an unufual fenfe of coldnefs in his thighs, and a palpable diminution of their fenfibility; that in a little time more, his limbs were frequently convulfed by involuntary twitchings, particularly in the night; that foon after this, he not only became incapable of walking, but that his power either of retaining or difcharging his urine and fæces was confiderably impaired, and his penis became incapable of erection.

The adult alfo, it is added, finds all the offices of his digeftive and refpiratory organs much affected, and complains conftantly of pain and tightnefs at his ftomach.

When a curvature is perceived either in an infant or an adult, it is generally attributed to a blow or ſome previous violence. Our author is of opinion, however, that this ſuppoſition is ſeldom, if ever, true in either caſe.

The true cauſe of this diſeaſe, he obſerves, is a morbid ſtate of the ſpine, and of ſome of the parts connected with it. He contends that in infants this is the ſole cauſe, and that external violence has nothing to do with it. In the adult, he will not aſſert that external miſchief is always out of the queſtion, yet he is perſuaded that the part in which it ſhews itſelf muſt have been previouſly in a morbid ſtate, as no degree of violence whatever is capable of producing ſuch an appearance as occurs in the diſeaſe he is treating of, unleſs the bodies of the vertebræ were by previous diſtemper diſpoſed to give way. In this diſtinction, we are told, conſiſts the very eſſence of the diſeaſe.

The true curvature is invariably uniform in being from within outwards; but it varies in ſituation, in extent, and in degree. In general the lower limbs alone feel the effect; but the author mentions five caſes, in one of which the arms only, and in the other four both legs and arms, were affected.

As

As the primary and ſole cauſe of all the miſchief, is a diſtempered ſtate of the parts compoſing, or in immediate connection with, the ſpine, tending and moſt frequently ending in a caries of the body or bodies of one or more of the vertebræ, no application made to the limbs themſelves, or ſuch remedies as electricity and cold bathing, can, as our author very juſtly obſerves, ever be of any poſſible uſe. The ſame failure of ſucceſs attends the uſe of the different pieces of machinery, all of which, from the moſt ſimple to the moſt complex, but particularly the ſwing and the ſcrew, are calculated, as we are told, to remove what does not exiſt. They are founded upon the erroneous ſuppoſition of an actual diſlocation, and therefore they always have been, and ever muſt be, unſucceſsful. They, who have had patience and fortitude to bear the uſe of them to ſuch a degree as to affect the parts concerned, have always found increaſe of pain and fever, and an exaſperation of all their bad ſymptoms, and our author has ſeen more than one inſtance in which the attempt has proved fatal. He takes this opportunity to caution his readers againſt the abſurd cuſtom of uſing theſe inſtruments to prevent growing children from becoming crooked, an an effect, he obferves, which, by forcing the fhoulders unnaturally backwards, they muft contribute to rather than prevent. If, inftead of adding to the embarraffment of childrens drefs by fuch iron reftraints, parents would throw off all of every kind, and thereby give nature an opportunity of exerting her own powers; and if in all cafes of manifeft debility refource was had to friction, bark, and cold bathing, with a due attention to air, diet, exercife, and the reft, the children of the opulent would, he thinks, ftand a chance of being as ftout, and as well fhapen, as thofe of the laborious poor.

In his former publication on this fubject the author was led to remark, that, previous to the appearance of the curvature, the general health of the patient does not feem to be materially, if at all affected. He very candidly acknowledges that a more enlarged experience in, and a more careful attention to the difeafe have convinced him that he was miftaken on this point; that moft, if not all the complaints of children, labouring under this infirmity, precede the curvature, and that a morbid ftate of the fpine, and of the parts connected with it, is the original caufe of both. An inference of the greateft importance may be deduced from this fact, as he

 is is ſatisfied that the malady may, in many inſtances, by early and proper attention, be prevented from producing its otherwiſe inevitable conſequences, temporary lameneſs, and permanent deformity.

In the ſame edition likewiſe he had deſcribed the bodies of the diſeaſed vertebræ, as being enlarged and ſpread; but upon repeated inquiry and examination, he is convinced that they are not, and that the diſeaſe does not ſo properly *enlarge* as erode. The ſtate alſo of the intervertebral cartilages, he finds to be ſubject to great variety, they being ſometimes totally deſtroyed, while the caries is ſmall in degree, ſometimes apparently but little injured, where the caries has done conſiderable miſchief, and ſometimes totally annihilated.

The remedy for this moſt dreadful diſtemper conſiſts merely in procuring a large diſcharge of matter, from underneath the membrana adipoſa on each ſide of the diſtempered bones forming the curvature, and in maintaining ſuch diſcharge until the patient ſhall have recovered his health and limbs. The idea of this mode of treatment, it ſeems, was firſt ſuggeſted to our author by the late Dr. Cameron of Worceſter, who who informed him, that, having remarked in Hippocrates an account of a paralyſis of the lower limbs, cured by an abſceſs in the back, he had, in a caſe of uſeleſs limbs attended with a curvature of the ſpine, endeavoured to imitate this act of nature by exciting a purulent diſcharge, and that it had proved very beneficial.

It is a matter, we are told, of very little importance towards the cure, by what means the diſcharge be procured, provided it be large, that it come from a ſufficient depth, and that it be continued for a ſufficient length of time.

Mr. Pott has tried different means of ſetons, iſſues by inciſion, and iſſues by cauſtic, and has found the laſt in general preferable, being leaſt painful, moſt cleanly, moſt eaſily manageable, and capable of being longeſt continued.

The cauſtics are directed to be applied on each ſide of the curvature, in ſuch a manner as to leave the portion of ſkin covering the ſpinal proceſſes of the protruding bones entire and unhurt, and ſo large, that the ſores upon the ſeparation of the eſchars may eaſily hold each three or four peas in the caſe of the ſmalleſt curvature; but in large curves, at leaſt as many more.

The iſſues are not only to be kept open, but the diſcharge from them is to be maintained by means of orange peas, cantharides in fine powder, ærugo æris, or any ſuch application as may beſt ſerve the intended purpoſe, which ſhould be that of a large and long-continued drain.

Whatever length of time it may take to obtain a complete cure, by reſtoring the health as well as the limbs, the iſſues, we are told, muſt be continued as long, and even a conſiderable time longer, eſpecially in the perſons of infants and growing children; the neceſſity of which, it is obſerved, will appear more ſtrongly, when it ſhall be conſidered, that infants and young children of ſtrumous habits are the ſubjects who are the moſt liable to this diſtemper, and that in all the time previous to menſtruation in one ſex, and puberty in the other, they are in general more ſerved by artificial drains than any other perſons whatever.

By means of theſe diſcharges the eroding caries is firſt checked, and then ſtopped; in conſequence of which, our author imagines, an incarnation takes place, and the bones unite and form a kind of anchyloſis.

Nothing,

Nothing, he obferves, can be more uncertain than the time required for the cure of this diftemper. He has feen it perfected in two or three months, and he has known it require two or three years; two thirds of which time paffed before there was any vifible amendment.

The firft fymptoms of amendment are defcribed to be a recovery of appetite, a return of refrefhing fleep, a more quiet and lefs hectical kind of pulfe, and if the patient is of an age to diftinguifh he will fay, that he has got rid of the diftreffing fenfation of tightnefs about the ftomach. In a little time more a degree of warmth, and a fenfibility is felt in the thighs, and generally much about the fame time, the power of retaining and difcharging the urine and fæces begins to be in fome degree exerted.

The firft return of the power of motion in the limbs is rather difagreeable, the motion being generally painful and fpafmodic, efpecially in the night. At this period of the cure, we are told, it is no uncommon thing, efpecially in bad cafes, for the patient to remain fome time without making any further progrefs; but in the milder kind of cafes, the power of voluntary motion generally foon follows the involuntary. The knees and ancles gradually lofe their their ſtiffneſs, and the patient is then able to ſet his feet flat upon the ground, which is the certain mark that the power of walking will ſoon follow.

The firſt attempts to walk are feeble, irregular, and unſteady; but when patients have arrived at this, our author has never ſeen an inſtance in which they did not ſoon attain the full power of walking. At firſt the patient finds it difficult to reſiſt, or to regulate, the more powerful action of the ſtronger muſcles of the thigh over the weaker, by which his legs are often involuntarily croſſed. Adults find aſſiſtance in crutches, &c. but the beſt and ſafeſt aſſiſtance for a child is ſaid to be what is called a go-cart, ſo made as to reach under the arms and encloſe the whole body.

The deformity remaining after recovery is ſubject to great variety. When one vertebra only is affected, and the patient young, the curve, we are told, will, in length of time, almoſt totally diſappear; but where two or three are affected, this cannot be expected.

In his former publication our author gave a ſhort account of the firſt two or three caſes which occurred to him. Theſe he has now omitted, becauſe the number of experiments which have been made by eminent practitioners, at home and abroad, have sufficiently eftablifhed the fact, and render the relation of particular cafes unneceffary.

The author informs us, that in the fpace of three years he has met with but one fingle inftance in which it has failed, where from the ftate of the difeafe, and of the patient, there was any reafonable foundation for hope; that all thofe who have fubmitted to keep the iffues open long enough, have been fo reftored to health, and to the free ufe of their limbs, as to be perfectly capable not only of exercife, but of hard labour, and that he has never yet, among thofe fo treated, met with one on whom the difeafe has returned.

Towards the clofe of the work the author gives us a variety of obfervations drawn from an attentive examination of the appearances that occur in this difeafe, and of their effects in different fubjects. He is convinced that the complaint arifes from a fcrophulous indifpofition, which fhews itfelf in a variety of forms; fometimes appearing in a thickened ftate of the ligaments of the fpine, at others in the form of a diftempered ftate of the intervertebral cartilages, or in that of difeafed glands. Sometimes times it is found in the form of bags or cyſts, containing in general partly a ſanious and partly a curd-like kind of ſubſtance. Sometimes under theſe bags or cyſts, even while they remain whole, the ſubjacent bones are found diſeaſed, and tending to become carious. In ſome ſubjects theſe collections erode the containing membranes, and make their way downward by the ſide of the pſoas muſcle, or by the ſide of the pelvis behind the great trochanter, or in ſome caſes to the outſide of the thigh.

Theſe different affections, we are told, are productive of many diſorders, general and local, of which, ſtrumous tubercles in the lungs, and a diſtempered ſtate of ſome of the abdominal viſcera often make a part.

When the ligaments and cartilages only, and not the vertebræ, are affected, the whole ſpine ſometimes gives way laterally, forming ſometimes one great curve to one ſide, and ſometimes a more irregular figure, attended with many marks of ill health.

When the attack is made upon the dorſal vertebræ, the ſternum and ribs, for want of proper ſupport, neceſſarily give way, and other deformity additional to the curve is thereby produced.

The

The author is perſuaded that this kind of caries is always confined to the bodies of the vertebræ, ſeldom or never affecting the articular proceſſes; that without this eroſive deſtruction of the bodies of the vertebræ, there can be no curvature of the kind which he is ſpeaking of, or, in other words, that eroſion is the *ſine quâ non* of this diſeaſe; that although there can be no curve without caries, yet there is, and that not unfrequently, caries without curve; that the caries with curvature and uſeleſs limbs, is moſt frequently of the cervical and dorſal vertebræ; the caries without curve, of the lumbal, though this is by no means conſtant or neceſſary; that in the caſe of carious ſpine, without curvature, it moſt frequently happens that internal abſceſſes are formed, and the matter either makes its way outward, or being detained within the body deſtroys the patient; that what are commonly called lumbal and pſoas abſceſſes are not unfrequently produced in this manner; and that a caries of the ſpine is more frequently a cauſe than an effect of theſe abſceſſes.

Five very accurate engravings are added, repreſenting the morbid appearances of the ſpine in different ſubjects.

